# A Sex Comparison of Fall and Fracture Occurrence in the Elderly Diabetic Population: A Quantitative Study

**DOI:** 10.1089/whr.2024.0158

**Published:** 2025-03-06

**Authors:** Fahamina Ahmed, Kaylin Miller, Meva Beganovic, Ali Siddiqui, Candice Smith

**Affiliations:** ^1^ Division of Clinical and Administrative Sciences, College of Pharmacy, Xavier University of Louisiana, New Orleans, Los Angeles, USA.; ^2^ College of Pharmacy, Xavier University of Louisiana, New Orleans, Los Angeles, USA.; ^3^ Louisiana State University Health, New Orleans School of Medicine, New Orleans, Los Angeles, USA.; ^4^ Ross University School of Medicine, Miramar, Florida, USA.

**Keywords:** falls and fractures in elderly patients with diabetes, sex comparison in falls and fractures, comorbidities

## Abstract

**Aims:**

To assess differences in falls and fractures in men and women with type 2 diabetes mellitus (T2DM) within a diverse population in Southeast Louisiana.

**Methods:**

A list of 1200 patients was generated through an electronic health record system using keywords: diabetic diagnosis, falls, and fractures to conduct this retrospective cohort study. This chart review included adults with T2DM who experienced at least one fall and/or fracture between January 2018 and May 2023 at East Jefferson General Hospital located in Metairie, Louisiana. Only falls and fractures that resulted in a hospital visit were included. Results were compared between males and females.

**Results:**

Patient data were collected from 100 randomly selected patients: 50 females and 50 males (mean age 67 years, 97% of patients were non-Hispanic, and 72% Black). Statistical analysis was conducted using the Student’s *t* test, Fisher’s exact, and Pearson correlation. An average of 3–4 falls occurred per patient, with no significant sex difference observed (*p* = 0.97). Thirty-eight percent of patients experienced a fracture with a significant sex difference (50% of female vs. 26% of male patients [*p* = 0.02]). Positive correlations between comorbid conditions and falls and fractures were seen, particularly in women: a moderate correlation for falls (*r* = 0.48, *p* < 0.01) and a strong correlation for fractures (*r* = 0.52, *p* < 0.01). Patients not on insulin treatment experienced a greater occurrence of fractures than insulin-dependent patients (46% vs. 22%, *p* = 0.03).

**Conclusions:**

Our findings suggest that in a diverse population, women with T2DM are at an increased risk of experiencing fractures, and specialized care should be given to this population to reduce the risk of fracture occurrence. Additional comorbidities increase the risk of falls and fractures.

## Introduction

Diabetes mellitus (DM) is a chronic health condition currently affecting 37.3 million people in the United States and over 422 million people globally.^1^ It is characterized by dysregulation of glucose, resulting in elevated blood glucose levels, predisposing to an array of adverse health conditions. There are three main types of diabetes, type 1 (T1DM), type 2 (T2DM), and gestational diabetes. T2DM is the most common, accounting for >90% of the cases.

Common health complications associated with diabetes are heart disease, chronic kidney disease, nerve damage, vision loss, and limb amputation.^2^ Individuals with diabetes have also been shown to have an increased risk of falls,^3–5^ particularly individuals who have tight glycemic control, with a hemoglobin A1C target below 7%,^3^ and those with uncontrolled diabetes.^6^ There have been mixed findings regarding sex differences and risk of falls, with some studies suggesting females are at an increased risk,^6^ whereas other studies did not have this finding.^7^

Diabetes has also been known to cause or exacerbate osteoporosis and osteopenia,^8^ placing patients at an increased risk for falls and fractures.^2,8,9^ Although individuals with T2DM have increased bone mineral density likely due to the protective factors of obesity, which is often comorbid with T2DM, they are still at an increased risk of developing osteoporosis and experiencing vertebral and hip fractures.^8,10,11,12^ Considering that diabetes-related fractures may lead to increased hospitalizations, financial burden, and decreased independence, it is vital to have a deeper understanding of the relationship between T2DM and fracture and fall prevalence.^13,14^

Previous studies have demonstrated a relationship between diabetes and the risk of experiencing falls and sustaining fractures. A study in Denmark found that individuals with diabetes had an increased risk of falls and fractures with increasing age and that females were more likely to experience falls.^15^ Many of the studies on falls and fractures have been conducted internationally and in predominately Caucasian populations. In our study, we were interested in exploring the sex differences associated with fall and fracture occurrence in a diverse, non-Caucasian population of individuals with diabetes in the United States.

In this retrospective study, we aim to assess the prevalence of fractures and falls in a diverse population of men and women with T2DM receiving care at East Jefferson General Hospital in Southeast Louisiana between January 2018 and May 2023. Identifying these numbers may lead to more preventative measures regarding fall and fracture risk in people with diabetes and bring awareness to a serious complication of diabetes.

## Materials and Methods

### Participants and data collection

The study follows a retrospective cohort design to compare the number of falls and fractures reported by men and women with diabetes. Data collection for the study began in February 2023. Inclusion criteria for the study included age 55 or older, diagnosis of diabetes, and at least one fall reported. A list of 1200 patients from East Jefferson General Hospital in Metairie, Louisiana, a community setting, was collected from the Epic electronic health record system that had keywords: diabetes diagnosis, falls, and fractures in their patient chart. A sample of 50 men and 50 women was selected randomly from the patient list by utilizing Epic and each patient’s medical record number. Data collected included demographic information including height, weight, body mass index (BMI), race, ethnicity, last reported hemoglobin A1C value, number of falls reported and their respective dates, number of fractures reported and their respective dates, and the number of comorbid conditions. In this study, a fall or fracture is identified as “reported” if the fall or fracture resulted in a hospital visit and was recorded in the patient’s chart between January 2018 and May 2023. The primary outcome was to compare the number of falls and/or fractures between women and men with a T2DM diagnosis. Secondary outcomes included a correlation for the incidence of fractures or falls for age, between comorbid conditions and sex, and insulin dependence. The study was approved by the institutional review board at LCMC Health–East Jefferson General Hospital.

### Statistical analysis

Stata 15 was used to analyze the study data. Descriptive frequencies were used to summarize patient characteristics. Student’s *t* tests and one-way analysis of variance were used to compare mean values between groups. Fisher’s exact tests were used to identify differences in outcome proportions among groups. Fisher’s exact test is an alternative to chi-squared tests that accounts for small sample size. *p* Values and Pearson correlation coefficients were used to determine the significance and strength of relationships between quantitative factors. Correlation coefficients were interpreted as follows: <0.3 as weak; 0.3–0.49 as moderate; and ≥0.5 as strong.

## Results

### Study population and demographics

The study sample included 100 patients, 50 females and 50 males. Table 1 details the demographic and clinical characteristics of the sample. In comparing female and male patients, there were no significant differences in demographics or mean clinical values. The average patient age was 67 years old; half of the patients (52%) were in their 60s. Most patients were non-Hispanic (97%) and Black (72%). The mean BMI was 30.8, with 72% of patients classified as overweight or obese. The mean hemoglobin A1C for the sample was 7.6, with patient values ranging from 4.8 to 14.6. The average number of comorbid conditions documented was 9.

**Table 1.** Baseline Demographic and Clinical Characteristics by Sex

		Sex		
	Total (*N* = 100)	Female (*n* = 50)	Male (*n* = 50)	*p* value
Age (mean, range)	67.2 (55–87)	68.5 (56–87)	65.9 (55–86)	0.089^a^
Age group (*n*, %)				0.476^b^
50s	15 (15%)	6 (12%)	9 (18%)	
60s	52 (52%)	24 (48%)	28 (56%)	
70s	24 (24%)	14 (28%)	10 (20%)	
80s	9 (9%)	6 (12%)	3 (6%)	
Ethnicity (*n*, %)				1.00^b^
Hispanic	3 (3%)	2 (4%)	1 (2%)	
Non-Hispanic	97 (97%)	48 (96%)	49 (98%)	
Race (*n*, %)				0.189^b^
Black	72 (72%)	40 (80%)	32 (64%)	
White	23 (23%)	8 (16%)	15 (30%)	
Other	5 (5%)	2 (4%)	3 (6%)	
BMI (mean, range)	30.8 (12.5–66.4)	32.3 (12.5–66.4)	29.3 (15.2–45.8)	0.120^a^
BMI category				0.561^b^
Underweight (12–18.4)	10 (10%)	5 (10%)	5 (10%)	
Healthy (18.5–24.9)	18 (18%)	10 (20%)	8 (16%)	
Overweight (25.0–29.9)	24 (24%)	9 (18%)	15 (30%)	
Obese (30.0 and higher)	48 (48%)	26 (52%)	22 (44%)	
A1c^c^ (mean, range)	7.6 (4.8–14.6)	7.7 (4.9–14.6)	7.5 (4.8–14.0)	0.737^a^
Number of comorbid conditions (mean, range)	9.1 (1–29)	9.6 (2–29)	8.6 (1–22)	0.357^a^
Insulin dependence	32 (32%)	16 (32%)	16 (32%)	1.00^b^

^a^ Student’s *t* test.

^b^ Fisher’s exact test.

^c^ Four patients have missing values for A1c.

BMI, body mass index.

### Primary data outcomes by sex and age

Table 2 outlines the number of falls, occurrence of fracture, and number of fractures by sex. While the mean number of reported falls and fractures was similar for female and male patients, the occurrence of fractures was significantly different. Half of female patients experienced fractures compared with only 26% of male patients (*p* = 0.023).

**Table 2.** Outcomes by Sex

		Sex		
	Total (*N* = 100)	Female (*n* = 50)	Male (*n* = 50)	*p* value
Number of falls (mean, range)	3.5 (1–24)	3.4 (1–24)	3.5 (1–13)	0.976^a^
Number of falls (median)	3.0	2.5	3.0	
Occurrence of fracture (*n*, %)				0.023^b^
No	62 (62%)	25 (50%)	37 (74%)	
Yes	38 (38%)	25 (50%)	13 (26%)	
Number of fractures (mean, range)	1.9 (1–10)	2.0 (1–10)	1.8 (1–5)	0.732^a^

^a^ Student’s *t* test.

^b^ Fisher’s exact test.

Table 3 outlines the number of falls, occurrence of fracture, and number of fractures by age groups. Except for patients in the 80s group, the mean number of falls and fractures reported increased with age. These increases, however, were not statistically significant. Also, the various age groups reported similar proportions of fracture occurrences.

**Table 3.** Outcomes by Age

	50s (*n* = 15)	60s (*n* = 52)	70s (*n* = 24)	80s (*n* = 9)	*p* value
Number of falls (mean, range)	2.2 (1–7)	3.5 (1–13)	4.5 (1–24)	2.8 (1–4)	0.192^a^
Occurrence of fracture (*n*, %)					
No	9 (60%)	34 (65%)	14 (58%)	5 (56%)	0.883^b^
Yes	6 (40%)	18 (35%)	10 (42%)	4 (44%)	
Number of fractures (mean, range)	1.0 (1–1)	1.4 (1–3)	3.0 (1–10)	2.5 (1–6)	0.096^a^
BMI (mean, range)	31.6 (18.9–46.4)	32.3 (12.5–66.4)	29.5 (17.8–43.0)	24.6 (17.4–36.6)	0.133^a^

^a^ Analysis of variance.

^b^ Fisher’s exact test.

BMI, body mass index.

### Correlations of comorbidities and primary outcomes

Table 4 outlines correlations between a number of comorbid conditions and primary outcomes. When analyzing the number of falls and comorbid conditions for all sampled patients, a weak correlation was found (*p* = 0.004). However, when analyzing the number of fractures and comorbid conditions for the 38 patients reporting fractures, a strong correlation was found (*p* = 0.001). Replicating this analysis by sex produced varying results. Significant correlations between comorbid conditions and falls/fractures were only found in female patients.

**Table 4.** Correlations with Number of Comorbid Conditions by Sex

		Sex	
	Total (*N* = 100)	Female (*n* = 50)	Male (*n* = 50)
Number of falls (*r*, *p* value)	0.28, 0.004^a^	0.48, 0.001^a^	0.01, 0.953^a^

	Total (*N* = 38)	Female (*n* = 25)	Male (*n* = 13)
Number of fractures (*r*, *p* value)	0.52, 0.001^a^	0.52, 0.007^a^	0.50, 0.083^a^

^a^ Pearson correlation.

### Insulin dependence and primary outcomes

Thirty-two patients in the study sample were insulin dependent. The proportions of insulin use among women and men were equal. The mean number of falls reported by patients using insulin was similar to those not using insulin (3.4 vs. 3.5). However, patients not using insulin experience a greater occurrence of fractures than insulin-dependent patients (46% vs. 22%, *p* = 0.03). There was no significant difference between insulin use and fracture/fall occurrence between men and women.

## Discussion

In this retrospective study, the findings indicate that, in our study population, men and women with T2DM have an equal prevalence of experiencing a fall, whereas women have a higher prevalence of sustaining a fracture. Our results support existing studies that have identified women with DM to have a higher prevalence of fractures than men.^6,15,16^ It has been postulated that these sex differences are due to changing hormone levels, with women losing bone mass rapidly in the first decade after menopause, attributed to declining estrogen levels.^17,18^

However, contrasting with population data, we found no significant sex difference in the average number of fractures per individual between women and men with diabetes. While more of our women participants sustained fractures overall, the number of repetitive fractures experienced by men and women was intriguingly similar. Considering that the average number of fractures per person was low (2 fractures) in both men and women, these findings may reflect lifestyle modification by women following an initial fracture, which decreases their likelihood of additional fractures.^19^ Our results support existing findings of falls and fractures increasing with age.^7^ However, in our study, patients in their 80s reported fewer mean falls and fractures than younger age groups. Possible explanations of this finding may reflect survivorship bias, whereby the healthiest subset of this advanced age group remains alive and ambulatory enough to access medical care. This is further supported by our findings that these participants had, on average, a healthy BMI score of 24. An alternative explanation is that individuals in their 80s may be less mobile, resulting in a lower risk of falls. Lastly, due to our low sample size of individuals in their 80s, we are unable to definitively draw conclusions from these results.

A history of falls can lead to the development of the psychological burden and a “fear of falling,” which may result in reduced activity, functionality, and self-care capability.^7^ Early prevention strategies, including mobility and balance exercises, are recommended to break this negative cycle in the aging population. In addition, individuals with diabetes commonly develop peripheral neuropathy and are two to three times more likely to have sarcopenia, making them vulnerable to injury.^20–22^ Physical exercise is essential to reducing the risk of falls and improving lower limb strength in these patients.

Our study also found significant positive correlations between comorbid conditions and both falls and fractures, particularly among women. Common conditions such as cardiac disease, hypertension, and depression amplify older individuals’ fall risk compared with those lacking such comorbidities.^7,19^ In addition, diabetes-associated comorbidities such as vision loss, peripheral neuropathy, arterial hypertension, orthostatic hypotonia, ischemic disease of the brain and heart, and lower extremities all increase the predisposition to falls.^23,24^ Our findings further this by illuminating increased comorbid disease burden associated with not only greater fall rates but also significantly higher fracture risks. Moreover, these relationships appear strengthened among female patients, suggesting women may suffer compounding declines as accumulating conditions intersect with diabetes-related bone health deterioration and age-related estrogen decline. Closely tracking coexisting illness patterns alongside sex could help stratify patients for tailored screening and early interventions to mitigate complication risks.

Our study found patients who did not use insulin for the treatment of their diabetes had more occurrences of fractures than those who used insulin. This result was not similar for the occurrence of falls, which is contrary to the result found in a meta-analysis that revealed the risk of falls was 162% higher for older adults with T2DM who use insulin.^25^ The use of insulin that may cause hypoglycemia^26^ or intensive control A1C ≤6 has been linked to a higher risk of falls.^27^ The role of polypharmacy and medications that may lead to falls in older patients has been studied extensively, indicating the same precaution is necessary for older patients with diabetes.

This study is limited by its small sample size from a single health system. Sample size was limited to 100 due to time constraints from the extensive charting time required to gather all relevant patient information. Broader multicenter studies would improve the generalizability of these findings and validate our observations. In addition, as a retrospective database review, information bias may affect injury reporting. The correlational analyses also limit causal inference about risk relationships. Other causal factors such as polypharmacy, polyneuropathy, or diabetic retinopathy were not evaluated, limiting alternative correlations or confounders related to the falls and fractures. The strengths of the study include analysis of factors such as BMI, A1C, comorbid conditions, and insulin dependence in a diverse population. However, this exploratory investigation provides impetus for deeper investigation into sex differences in diabetic bone health.

## Conclusion

Our findings suggest that in a diverse population, women with T2DM are at an increased risk of experiencing fractures. Considering the health, financial, and independence implications that fractures may present, particular care and preventive strategies should be given to this population to reduce the risk of fracture occurrence. In addition, a correlation between the number of health comorbidities and fracture and fall occurrence was observed, suggesting an increased risk of falls due to a compounding effect. Future studies may consider assessing lifestyle factors including diet, exercise, and alcohol and smoking consumption, and their impact on falls and fractures in this population.

## Abbreviations Used

A1C: Glycated hemoglobin
BMI: Body mass index
DM: Diabetes mellitus
T1DM: Type 1 Diabetes mellitus
T2DM: Type 2 Diabetes mellitus
